# Gonadotropin-releasing hormone receptor (*Gnrhr*) gene knock out: Normal growth and development of sensory, motor and spatial orientation behavior but altered metabolism in neonatal and prepubertal mice

**DOI:** 10.1371/journal.pone.0174452

**Published:** 2017-03-27

**Authors:** Ellen R. Busby, Nancy M. Sherwood

**Affiliations:** Department of Biology, University of Victoria, Victoria, BC, Canada; John Hopkins University School of Medicine, UNITED STATES

## Abstract

Gonadotropin-releasing hormone (GnRH) is important in the control of reproduction, but its actions in non-reproductive processes are less well known. In this study we examined the effect of disrupting the GnRH receptor in mice to determine if growth, metabolism or behaviors that are not associated with reproduction were affected. To minimize the effects of other hormones such as FSH, LH and sex steroids, the neonatal-prepubertal period of 2 to 28 days of age was selected. The study shows that regardless of sex or phenotype in the *Gnrhr* gene knockout line, there was no significant difference in the daily development of motor control, sensory detection or spatial orientation among the wildtype, heterozygous or null mice. This included a series of behavioral tests for touch, vision, hearing, spatial orientation, locomotory behavior and muscle strength. Neither the daily body weight nor the final weight on day 28 of the kidney, liver and thymus relative to body weight varied significantly in any group. However by day 28, metabolic changes in the GnRH null females compared with wildtype females showed a significant reduction in inguinal fat pad weight normalized to body weight; this was accompanied by an increase in glucose compared with wildtype females shown by Student-Newman-Keuls Multiple Comparison test and Student's unpaired t tests. Our studies show that the GnRH-GnRHR system is not essential for growth or motor/sensory/orientation behavior during the first month of life prior to puberty onset. The lack of the GnRH-GnRHR axis, however, did affect females resulting in reduced subcutaneous inguinal fat pad weight and increased glucose with possible insulin resistance; the loss of the normal rise of estradiol at postnatal days 15–28 may account for the altered metabolism in the prepubertal female pups.

## Introduction

Gonadotropin-releasing hormone (GnRH) is an essential neuropeptide in the onset and control of reproduction. However, GnRH may also affect non-reproductive processes as suggested by its expression long before puberty in the fetal period [1, 2] and by the widespread presence of GnRH receptors (GnRHR) outside of classical reproductive tissues [3, 4].

The role of GnRH in novel, non-reproductive processes during postnatal development could be a direct central effect on brain neurons that contain GnRH receptors or a direct peripheral effect of GnRH synthesized in peripheral tissues acting on local GnRH receptors. It is unlikely that direct effects are due to GnRH in circulating blood as its concentration is very low except for portal blood that perfuses the pituitary. Also, GnRH could act indirectly by releasing the gonadotropins LH and FSH, which in turn, release sex steroids known to affect the brain and peripheral tissues. These downstream hormones that are activated by GnRH are also expressed early in development; LH, was detected in the fetal mouse pituitary at embryonic day (E)14-18 and in plasma at E16-18 [5,6]; the LH receptor (LHR) was found in the testis at E16 [5, 7] suggesting that the GnRH-GnRHR system is a candidate for indirectly affecting central or peripheral targets before puberty.

The non-reproductive tissues or processes that might be directly affected by GnRH are limited to those with a GnRH receptor. There is widespread distribution of GnRH and this has been reviewed [3]. Neurons containing GnRH1 were found in the anterior hypothalamus, medial preoptic area, ventral telencephalon including the medial septum-diagonal band, and in the olfactory system including the terminal nerve ganglia in rats [8]. This group of neurons is associated with reproductive functions in that many of their axons release GnRH into the portal blood for delivery to the pituitary. More relevant to the present study, many of the GnRH axons or branches do not terminate in the median eminence, but rather in other brain areas, In mammals, GnRH cell bodies or axons have been detected in the cerebellum, piriform cortex, cerebral cortex (low levels), hippocampus, midbrain central gray, spinal cord, tissues surrounding the brain ventricles and in the retina [3, 8, 9, 10]. Additionally, GnRH1 has been detected in the cerebrospinal fluid [9] making it another source of peptide for binding receptors in the brain. Many of these regions in the central nervous system affect motor, spatial or sensory functions. GnRH2 is found in the midbrain region, except in mice and other mammals that have lost or silenced the *Gnrh2* gene [11, 12]. Outside of the brain, GnRH1 has been detected in the pituitary and other reproductive organs including the ovary [13–15], placenta [16, 17], testis [15, 18, 19] and prostate [20, 21], but also in non-reproductive organs including the liver, heart, kidney, skeletal muscle [17], bone marrow, blood mononuclear cells [22, 23] and incisor teeth [24]; GnRH2 has been detected in prostate, bone marrow and kidney in humans [25]. Although GnRH1 in peripheral tissues is known to produce direct local effects, [14, 16, 21], it is the direct central effects that are considered here to be important in the development of motor and sensory systems. The indirect effects of GnRH via the sex steroids may also affect prepubertal development and metabolism of these systems.

Likewise, receptors for mammalian GnRH have been reported to be widespread in the brain including areas related to motor control and sensory detection: frontal cortex, cingulate cortex, cerebellum, superior colliculus, red nucleus, thalamus, dorsal hippocampus, amygdala and central gray of the midbrain, interpeduncular nucleus, spinal cord [10, 26, 27] and olfactory-related areas including the piriform cortex, anterior olfactory nucleus, olfactory tubercle, olfactory bulb and terminal nerve [3, 8, 27]. GnRHRs that are in the eye and retinal neurons [3, 8, 26] may play a role in special sensory functions. In areas related to neuroendocrine control of reproduction, GnRHRs are found in the hypothalamus (arcuate and ventromedial nucleus), preoptic area, septum, amygdala and habenula [3, 8, 28]. Outside the brain, GnRH receptors are found in both reproductive tissues such as the anterior pituitary [3, 8, 28–30], ovary [29], placenta [16, 28], testis [18, 19, 30], and in non-reproductive tissues such as heart, kidney, liver, bladder, skin, skeletal muscle, adrenal, lymphocytes and some cancer cells [3, 17] providing a number of potential targets for GnRH. However, evaluation of the importance of GnRH and GnRHR outside of the reproductive axis is difficult to measure; quantitative measurements of mRNA and protein expression are helpful but do not provide evidence that the GnRH-GnRHR system has identifiable functions *in vivo* or *in vitro* outside of the reproductive axis. In summary, the presence of GnRH and its receptor are sufficiently distributed to suggest a direct GnRH action could occur in the CNS to affect the development of motor and sensory functions during the first month of life in mice.

To address the question of whether GnRH affects non-reproductive functions before puberty, we examine mice in which the GnRH receptor is disrupted (GnRHR knockout) during the prepubertal period. The GnRHR-null mice produce normal GnRH, but both the direct and indirect pathways of GnRH activation are blocked. GnRH cannot act directly due to the lack of its receptors in central neurons or peripheral tissues nor can it act indirectly due to the lack of its pituitary receptors that release FSH/LH needed for downstream release of sex steroids. If significant changes occur in non-reproductive functions of GnRHR-null mice during development, then it would require further studies to determine if the changes are direct or indirect actions. However, if changes are not detected, then it is clear that neither direct nor indirect actions of GnRH are important for the non-reproductive processes studied in this mouse line with a global receptor knockout.

In regard to indirect effects of GnRH, the postnatal period between birth and puberty is no longer considered to be an entirely quiescent period in regard to the GnRH-GnRHR-FSH/LH- sex steroid system. GnRH does not affect differentiation of the testis during gestation, but GnRH is known to be essential for the sexual differentiation of the brain. Kisspeptin-induced GnRH releases gonadotropins, which stimulate a brief testosterone surge that feedsbacks on the male brain to initiate sexual differentiation (masculinisation). [31]. Thereafter, the level of testosterone remains low (0.04 to 0.6ng/ml) during postnatal days P2-P30 [31–34]. It is this period in males that offers a window to study relatively direct actions of GnRH on the nervous system and other organs.

GnRH affects the female brain in a different way compared with the male during the period before puberty. In female pups at P15, estradiol begins to rise either due to increased output from the ovary or to a reduced amount of α-fetoproteins that bind estradiol [35, 36]. The female brain is considered to be feminized beginning at P15 resulting in adult female sexual behavior. The proof is substantial [37], although indirect because measurement of estradiol at any stage has technical problems [38, 39]. To determine whether the non-reproductive processes are affected by male or female sexual differentiation of the brain or by the rise in estradiol at P15-P22, different genotypes (WT vs null) in the *Gnrhr* KO line were compared.

To select a mouse line in which the *Gnrh* receptor gene is knocked out (*Gnrhr* KO), it is important that the line has a complete loss of the GnRH-GnRHR signalling system from the time of conception. Previously we prepared a *Gnrhr* KO mouse using the gene trap method. The phenotype was invariant, including lack of primary and secondary sexual organ development, low levels of FSH, LH and sex steroid hormones, and infertility including a lack of sexual behavior [40]. This severe form of hypogonadotropic hypogonadism resulted from disruption of the type 1 *Gnrhr* gene, the only type retained in the mouse genome. This mouse line has sex differentiation of the gonads, but lacks sexual differentiation of the brain in males and females [31, 40].

Another *Gnrhr* KO model is the mouse line in which the receptor has a single amino acid substitution (L117P) resulting from an ENU-induced mutation [41]. This mouse line showed a severe form of hypogonadotropic hypogonadism, but less severe than the gene trap *Gnrhr* KO mouse described above. A third mouse line with a disruption of the GnRH receptor (E90K) had only a mild phenotype [42]. Non-reproductive effects, except for body or brain weight, were not studied in the three lines of defective GnRHR mice.

The hypogonadal (*hpg*) mouse, which has a defective *Gnrh* gene [43], is not suitable to study the effect of GnRH loss on non-reproductive functions because the fetal *hpg* mouse has intact GnRH receptors that could respond to normal transplacental GnRH, which the heterozygous mother produces. Meanwhile, the model with loss of the GnRH receptor from the time of conception [40] would exclude this concern.

In the present study, mice lacking GnRH receptors were examined in regard to early development (the first month of life after birth) for behavior, growth and metabolism. Behavior included development of locomotion, muscle strength, spatial orientation, and sensory response to auditory, tactile and visual stimuli. Growth included weight of the total body and individual organs. Metabolism included measurement of glucose and analysis of changes in weight and histology of the adipose tissues. The *Gnrhr* KO model is used to determine if a null mouse that is not exposed to the effects of GnRH from conception has non-reproductive developmental defects in the first month of life.

## Materials and methods

### Mouse line with *Gnrhr*-targeted disruption

In our mouse line, the *Gnrhr* gene was disrupted by the gene trap method as previously described [40]. As the null mice do not mate or reproduce, heterozygous male and female mice were used as breeders resulting in an overall 1:2:1 ratio of wild type (+/+), heterozygous (+/-) and homozygous (-/-) offspring. Genotyping involved earclips and three specific primers for PCR as described [40].

### Animals

All procedures were approved by the Animal Care Committee at the University of Victoria (Protocol Number 2011–001). Humane endpoints were in place during this animal study in that specific clinical criteria were established for euthanasia as outlined in our protocol. In Experiment I, heterozygous *Gnrhr* C57BL/6J female mice were mated with a heterozygous male who remained in the cage for about one week, but was not present when the pups were born or reared. Birth was considered to be Day 0 and all litters were size adjusted within the first 2 days to 7 or 8 pups. Each mother with her pups was housed in a plastic cage (19 x 30 x 19 cm) with corn cob bedding along with nesting bedding and a plastic "igloo" house for enrichment. The pups remained with the mother until the final test on Day 28. Behavioral testing occurred in the morning from Day 2 through Day 28. Laboratory rodent diet with 6% fat (Lab Diet 5001, PMI Nutrition International) and tap water were freely available at all times. Lights were on for 12 hours from 0600 in a 23°C room.

In Experiment II, conditions were the same except that the mice were not disturbed for behavioral testing. The average number of pups was 7.2 per litter. The pups were removed on Day 28, weighed and anesthetized for collection of blood, fat pads and organs.

### Experimental design for behavioral testing in Experiment I

#### Transfer and marking

Four pups at a time were randomly selected from the home cage and transferred to adjacent rooms for marking, weighing and testing. On Days 2–10, each mouse was daily marked with a number from 1–8 on its abdomen using an indelible nontoxic marker (VWR, fine tip) and as a double check, the tail was coded with nontoxic colors using a pen (Staedtler permanent lumocolor). On Day 11, the pups were ear clipped as fur prevented further marking on the abdomen but tail color coding was continued until the end of the experiment on Day 28.

Within ten minutes of removal from the mother, the pups were transferred to a heated (30° - 35°C) holding cage with corn cob bedding. The temperature was continually monitored by a small probe on top of the bedding. Each pup was individually tested for up to 10 min at 27–32°C and then returned to the holding cage. The four pups were returned to the mother at one time, while the other four pups were removed for the same routine.

#### Body weight and fur development

As a general measure of health, each pup was weighed individually on Days 2 to 28. Also, each mouse was observed for the initial appearance of fur on the head and back. During the following days, hair appeared on the sides of the body and abdomen and was observed for any roughness or patchiness.

#### Sequence of behavioral tests

After the pups were marked and weighed, their tests were given in the following order: 1) presence of eye opening and fur, 2) locomotion, 3) "negative geotaxis" or reorientation on an inclined plane, 4) muscle strength on horizontal and vertical mesh screens, 5) cliff avoidance, 6) grasp reflex, 7) bar hanging, 8) auditory startle, 9) tactile startle and 10) righting reflexes. The most strenuous tests were the vertical screen climb, bar hanging and righting reflexes, so less demanding tests were given between the climbing and hanging tests; air righting was performed last due to the stressful nature of the test. A maximum of two minutes was required for weighing and marking and ten minutes for testing.

### Behavior related to sensory development

#### Grasp reflex in response to touch

Pups were gently held vertically and the palm of each forepaw was touched with an 18-gage stainless steel tube. The reflex was recorded as positive if the pup curled its digits around the tube.

#### Tactile startle

A puff of air was applied to the hind quarter of the pup without touching the pup. The air puff was produced by squeezing a baby's rubber suction bulb device. A rapid startle response by the pup was recorded as positive.

#### Tactile cliff avoidance before eye opening

Each pup was placed on a smooth white platform that was 20 x 20 cm and 50 cm above the bench on a stable stand. Soft foam rubber was on the bench below the platform. The pup was placed with its forepaws just over the edge of the platform and observed as to whether it moved back from the edge with one or both paws. The ability of the pup to stay on the platform whether quiet, pivoting, crawling or walking for 30 seconds was recorded for days 2–28.

#### Eye opening

Eyes were examined daily for opening. The first day in which an eye began to open (slit) or was wide open was recorded separately for the left and right eye.

#### Visual cliff avoidance

Pups were observed as to whether they tested the edge of the elevated platform and pulled back without falling during a 30 second period. Since pups can walk before their eyes open, some walked off the platform and were caught. The first day in which the pups did not walk off for three days in a row was recorded and noted to coincide with eye opening.

#### Auditory startle

One click of a dog training clicker was applied above and behind the mouse resting on the bench. A rapid startle response (usually a jerk or contraction) was considered positive.

### Behavior related to development of spatial orientation

#### Orientation on an inclined plane (formerly "negative geotaxis")

Each pup was placed on an inclined plane with its nose pointing downwards. The plane (20 x 20 cm) was at a 30° angle from the horizontal plane and covered with 16-mesh wire screen. The latency period (seconds) was recorded for the pup to change its orientation so that its head faced up the incline. A maximum of 60 secs was allowed [44]. The first day in which the mouse completed a 180° rotation in less than 8 sec was used for comparison. The best performance was during the early stage in which the mouse could pivot, but was not able to crawl or walk away in another direction. The apparatus was placed on a covered foam rubber mat.

#### Righting reflex from a surface

The pup was placed onto its back on a flat surface and the time (seconds) was recorded for the mouse to turn over onto its abdomen with all four legs under the body. A maximum of 60 seconds was allowed for the test. The first day in which the pup righted itself within one second was used for comparison.

#### Righting reflex in air

The pup was held with two hands so that the back of its head and body were toward the bench surface below. The pup was dropped from a height of 30 cm above a soft foam rubber pad. The mid-air righting occurred in less than a second. The first day in which the pup righted itself in air and landed on all four feet was used for analysis.

### Behavior related to development of locomotion

#### Elevation of head and shoulders

Each pup was placed on a smooth white surface (20 x 20 cm), which was directly on the bench. The ability of the pup to lift its head and shoulders was observed for a two minute period.

#### Pivoting

As above, each pup was observed for its ability to pivot, which occurred in pups that elevated the head and shoulders while turning but were unable to use their hind legs.

#### Crawling

As above, each pup was observed for its ability to crawl. Crawling was defined as the ability to move forward or backward using all four limbs without lifting the abdomen from the surface.

#### Walking

The day was recorded when the pup was able to lift its body above the surface and walk with its four limbs in a forward direction for at least two steps during the 2 minute observation. If the pup didn't move for 30 seconds and may have gone to sleep, it was gently lifted into the air and replaced on the platform. Beginning at 10 days of age, the mice were placed in an open cage (19 x 30 x 19 cm) for observation of walking. Rearing and grooming were not analyzed as most pups were rearing and washing in their holding cage before day 10.

### Behavior related to muscle strength

#### Level screen resistance

The pup was placed on a level metal screen (16 mesh) that was 28.5 x 28.5cm with a smooth perimeter. The pup was gently pulled by its tail in a horizontal direction across the screen to determine whether the pup could hold onto the screen and resist the pulling.

#### Bar hanging

Each pup was gently lifted by its trunk so it could grab a metal bar (1mm diameter for pups up to 10 days of age and 2mm diameter for pups from 11–28 days). Each bar was permanently held in a separate frame so that the bar was 18cm long and 25cm above the bench; the smooth sides of the frame extended well beyond the bar so the mouse could not grab the frame in any direction. After the pup had grabbed the bar with its forepaws, the observer's hand was held below to catch the pup when it released from the bar. A maximum time of 10 seconds was allowed for hanging. Both the first day in which the pup held onto the bar for 10 sec and the first day in which the mouse repeated the 10 sec hang for three consecutive days were recorded. Many mice preferred grabbing the bar when they were upside down; the observer then moved the mouse to an upright (hanging) position. The tail was gently held down when a mouse tried to put its hind foot over the bar.

#### Vertical screen climb

Each pup was placed near the bottom of the level screen. The screen was then quickly rotated from a horizontal to a vertical position with the mouse's nose pointing upward. The ability of the mouse to start climbing up the vertical screen by at least two steps within 60 sec was observed. A foam rubber pad was placed below.

### Growth of kidney, liver, thymus and fat pads in Experiment II

On day 28 or 29, each mouse was anesthetized with isofluorane. Initially, cardiac puncture with a heparinized syringe and 23 gage needle was carried out through the closed chest. Immediately after, the mouse was surgically opened and the kidney, liver and thymus were removed and weighed. The four fat pads (inguinal, gonadal, retroperitoneal and scapular) were dissected and weighed individually. Gonadal fat pads were placed in Bouin's fixative solution.

### Glucose measurement in blood removed by cardiac puncture in Experiments I and II

Immediately after the blood withdrawal, a drop or two of blood was used to determine glucose levels. The remaining blood was transferred to a 4.0 ml heparinized tube (BD Vacutainer with 75 USP units of sodium heparin from Becton, Dickinson & Co., Franklin Lakes, NJ) for centrifugation. The plasma was transferred to an Eppendorf tube and stored at -80°C. For glucose determination, a Contour glucometer (Bayer Inc., Toronto, ON) was used. In Experiment II, in which the mouse had not been handled, glucose measurements were in duplicate. In Experiment I, in which the mice were tested for behaviour on days 2–28, single measurements were recorded unless a high or low value suggested a duplicate was needed.

### Mortality

In Experiment I, mortality was 2.7% (2 out of 74 pups). One pup died on day 19 and all data were excluded as the pup began to lose weight on day 14. The other pup did not die until day 25 so data through day 20 was included as the mouse was still gaining weight on day 24. One mouse had microphthalmia, so its data on eye opening were not included.

### Histology of white adipose tissue

The gonadal fat pads from three mice in each category of male and female *Gnrhr* (+/+), (+/-) and (-/-) genotypes were fixed in Bouin's solution. Thereafter the tissue was dehydrated through a graded series of ethanol to 100%, then embedded in JB-4 plastic (Electron Microscopy Sciences, Hatfield, PA). The tissues were sectioned at 5μm thickness with a JB-4 microtome and stained with hematoxylin and eosin. Images were imported into Open Lab software.

### Statistical analysis

Data are expressed as the mean ± SEM for each group. Multiple comparisons were analyzed by one-way ANOVA, followed by *post hoc* comparisons with Student-Newman-Keuls Multiple Comparison test. Because of the importance of comparing the wildtype and null mice, Student's unpaired t test was used for the comparison of mean differences between two different genotypes of the same sex (male wild type (+/+) versus male null (-/-) or female (+/+) versus female (-/-). Prism statistics software (GraphPad Inc., San Diego, CA) was used for statistical analyses of data. All data are shown as the mean ± SEM. Significance of differences was considered to be P < 0.05.

## Results

### Experiment I. Pattern of weight gain in developing pups lacking GnRH receptors

Parents that were both heterozygous for the *Gnrhr* gene produced viable male and female pups of three genotypes: *Gnrhr* +/+ (wildtype, WT), +/- (heterozygous, het), and -/- (homozygous or null) in the expected Mendelian ratio of 1:2:1. Developmental anomalies in the offspring were not apparent externally on days 2–28 except for secondary sexual characteristics, which were poorly developed as shown in *Gnrhr* null males by a small penis and short anogenital distance.

**Fig 1:**
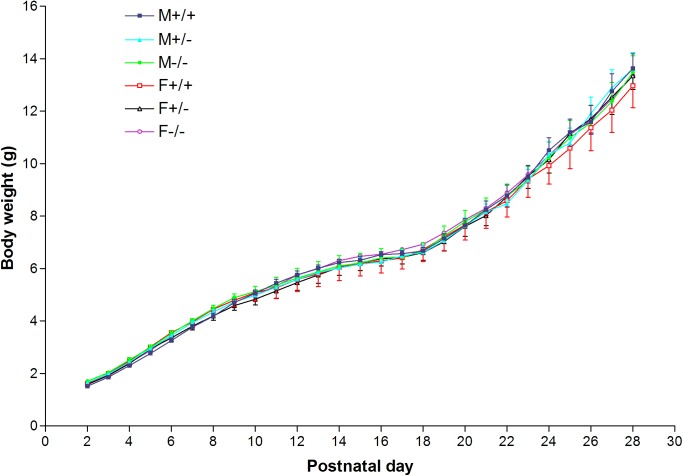
Body weight on postnatal days 2–28 for a line of mice lacking the *Gnrhr* gene. No significant difference was present on any day with Student-Newman-Keul’s Multiple Comparison test for the six groups or with the Student’s unpaired t-test for null versus wildtype for each sex. (F = female; M = male; +/+ = wildtype; +/- = heterozygous; -/- = null).

The body weights for the three genotypes of both males and females were recorded daily for days 2–28 (Fig 1). The pups remained with their mother throughout this period in litters of 7 or 8 pups. The body weight did not vary significantly on any day for the six groups of mice (male WT, het, null and female WT, het, null), although each mouse was tested and/or handled daily for about 12 minutes. Statistical analysis by the Student-Newman-Keuls (SNK) multiple comparison test of the body weight each day for the six groups did not show any significant difference (p > 0.05) on any day. The most important groups (+/+ versus -/-) were further tested by sex with t-tests using male +/+ versus male -/- or female +/+ versus female -/-; none were significantly different (p>0.05).

### Experiment I. Development of responses to sensory input in *Gnrhr* null mice

**Fig 2:**
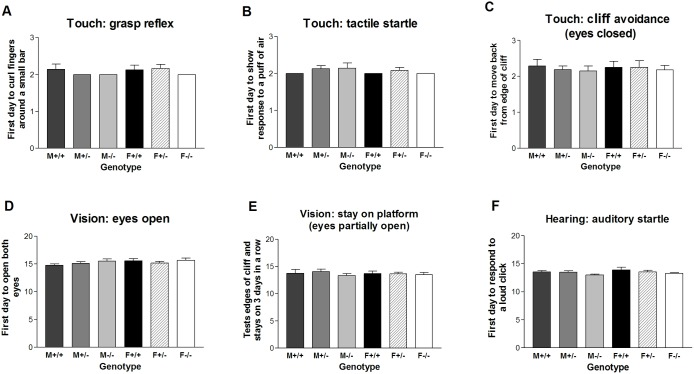
Sensory development for a mouse line with disruption of the GnRH receptor (*Gnrhr*) gene. Sense of touch is indicated by response to (A) grasp reflex, (B) tactile startle and (C) cliff avoidance by retreat from tactile sensing of edge. Vision was measured by (D) eye opening and (E) cliff avoidance by visual sensing of edge. Hearing was measured by auditory startle (F). (M = male; F = female; +/+ = wildtype; +/- = heterozygous; -/- = null).

The response of *Gnrhr* null mice to touch was evaluated in three different tests. The grasp reflex was present in the wildtype males and females on day 2, the first day of testing. Neither the heterozygous nor null groups were significantly different from the WT on the first day to show a grasp reflex (Fig 2A; group mean values 2.0–2.2 days). The same pattern was shown for all six groups for the tactile startle test; pups were capable of responding to a puff of air on day 2 with no significant difference (p>0.05) among the groups for genotype or sex (Fig 2B; group mean values 2.0–2.1 days). A final confirmation that 2-day-old pups respond to touch was exhibited in the cliff avoidance test. The eyes had not opened in any pups and yet they moved back as soon as their forepaws were placed over the edge of a cliff, which was 50 cm above the bench. During the next 30 sec. the pups remained quiet or pivoted as they had on the bench. Occasionally a pup rolled or pivoted off the platform due to lack of eye sight and motor coordination; all pups who fell were caught. Significant differences did not occur between the six groups (Fig 2C; group mean values 2.1–2.3 days).

Mice are born at an early developmental stage in which the eyes do not open for about two weeks. To determine the exact age at which the C57BL/6J mice lacking a GnRH receptor open their eyes as a sign of development, the day for a slit opening in one eye or for full opening in both eyes was recorded. The first day to open both eyes occurred at group mean values of 14.7–15.6 days in all groups without significant (p>0.05) differences (Fig 2D). Vision is likely to have been sufficient to avoid the cliff for a few days before both eyes were fully open as the mean of each of the six groups ranged from 13.3–14.1, which represented the first day when the pup stayed on the platform without falling for three consecutive days (Fig 2E). These data strongly support normal development of vision in mice lacking a GnRH functional system from the time of conception.

Hearing also developed normally in *Gnrhr* null mice as evaluated by their auditory startle response to a loud click behind them. Group mean values of 13.0–13.9 days in the six groups of male and female pups of three genotypes showed no significant difference among the groups; t-tests confirmed this result for wildtype versus null mice (Fig 2F).

**Fig 3:**
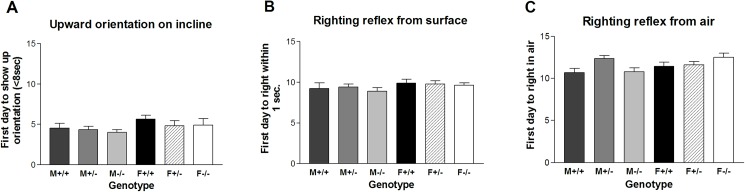
Spatial orientation development in a mouse line lacking the *Gnrhr* gene. The figure shows the first day in which mice (A) oriented upward on an incline; (B) completed a righting reflex from a flat surface; (C) developed a righting reflex from the air. (F = female; M = male; +/+ = wildtype; +/- = heterozygous; -/- = null).

“Negative geotaxis” was thought to be a response to gravity in pups but is now considered to be a postural response related to spatial orientation or improving stability [45] in which a mouse that is placed with its head pointed down a ramp will turn until the head points directly up the incline. The vestibular organ is involved in this response. On day 2 the pups already attempted to pivot on the screen to orient the head up the incline, but the rotation tended to be slow or incomplete. However, the mean value for the six groups ranged from 4.0 to 5.7 days for the mice to complete the rotation in less than 8 seconds. Thereafter, many mice that were crawling or walking moved in another direction with less than 180° rotation. Significant differences did not appear in comparisons within the six groups or in the t-tests (Fig 3A).

The development of righting reflexes related to spatial orientation was examined by the time taken for a righting reflex when the back of the pup was either on the bench surface or in the air. Righting involves a response to sensory information from proprioceptors in the joint capsules, vestibular receptors, and cutaneous touch and pressure receptors [46]. Because pups initially turn over or right themselves incompletely from the surface with one hind leg not tucked under the body, the first day was defined as the one in which righting was completed with all legs under the body in less than one second. The air reflex was evaluated as the day in which the mouse landed on four feet. The *Gnrhr* null mice were able to perform these reflexes on the same developmental schedule as the wildtype and heterozygous mice (SNK p>0.05; t-tests >0.05). The mean value for the first day to right from the surface was 8.9 to 9.9 for the six groups and from air was 10.7 to 12.5 days (Fig 3B and 3C).

Thus, mice lacking GnRH receptors were not affected in their ability to detect a number of sensory inputs through different receptors and respond with an appropriate physical reaction.

### Experiment I. Development of locomotory behavior and muscle strength in *Gnrhr* null mice

**Fig 4:**
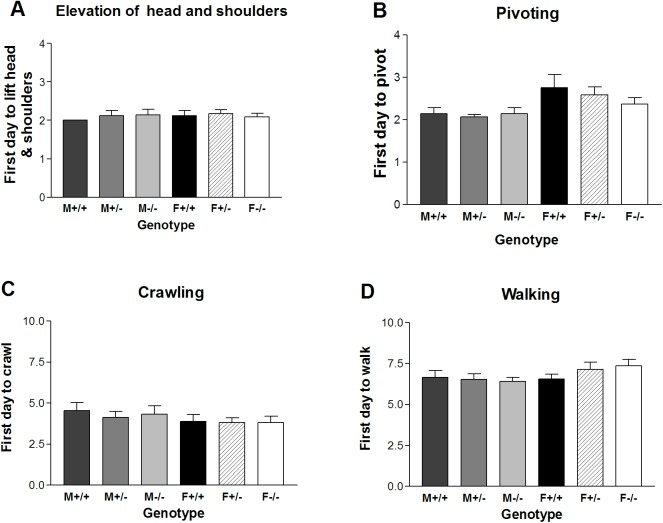
Locomotor development in a mouse line with disruption of the *Gnrhr* gene. The six groups of mice, including males and females, wildtype (+/+), heterozygous (+/-) and null (-/-) groups, were tested for the first postnatal day in which (A) the head and shoulders could be elevated; (B) the mouse could pivot; (C) the mouse crawled and (D) the mouse walked. (F = female; M = male).

*Gnrhr* null mice of both sexes were able to lift the head and shoulders on the first day of observation. The mean values for the six groups ranged from 2.0 to 2.2 days and lacked statistical difference (SNK p>0.05, t-test p>0.05) (Fig 4A). Pivoting followed soon after the pups were able to elevate their head and shoulders with a similar developmental pattern in which there was not a significant difference in the mean values of the six groups, 2.1–2.8 days (Fig 4B).

Crawling commenced on developmental days 3.8 to 4.6 in the six groups without significant statistical differences (Fig 4C). Walking was clearly distinct from crawling as the pups were strong enough to lift the abdomen off the surface and move forward. It was a dramatic change with a clear endpoint that did not differ significantly among the six groups beginning on days 6.4–7.4 (Fig 4D).

**Fig 5:**
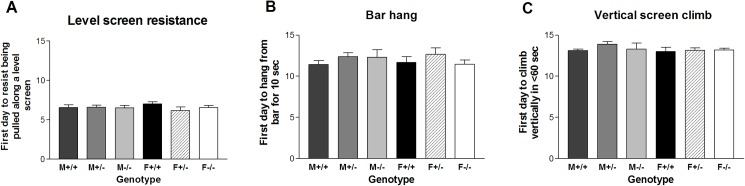
Muscle strength development in a mouse line with disruption of the *Gnrhr* gene. Mice were tested for the first postnatal day in which they (A) resisted being pulled along a level screen; (B) hung from a bar for 10 sec; (C) climbed up a vertical screen in less than than 60 sec. (F = female; M = male; +/+ = wildtype; +/- = heterozygous; -/- = null).

Muscle strength was evaluated by three different tests: level screen resistance, bar hanging and vertical screen climbing. Younger pups developed the ability to resist being pulled across a horizontal screen; the *Gnrhr* null pups did not differ significantly (SNK, t-tests) from the WT or heterozygous mice as shown by the six group means ranging from 6.2 to 7.0 days (Fig 5A). Older pups were able to do bar holding (mean values of 11.4 to 12.7 days for the six groups) and later to climb up a vertical screen (13.0 to 13.9 days for the six groups); the null pups developed the same pattern for muscle strength compared with the other genotypes without significant differences (Fig 5B and 5C). At these ages in the range of 6–14 days, even the males and females did not differ on these strength tests that require muscle development.

### Experiment II. Effect of handling during behavioral testing of pups

**Fig 6:**
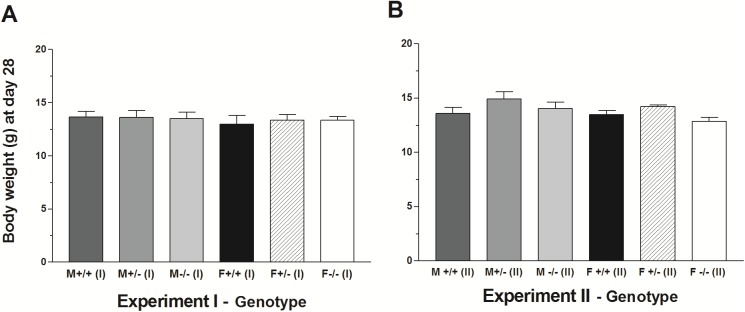
Comparison of body weight for handled and non-handled 28-day-old mice. Body weights (mean±SEM) are compared for effect of handling in (A) experiment I (daily testing) with (B) experiment II (no testing) in *Gnrhr* male and female genotypes. (F = female; M = male; +/+ = wildtype; +/- = heterozygous; -/- = null).

The behavioral tests require handling each pup every day for about 12 minutes on days 2–28. To evaluate whether this routine might stress the pups and cause loss of body weight, a separate experiment with six groups that matched those subjected to behavioral tests was performed. The pups were left with their birth mother and not handled except for routine change of bedding. The result of statistical analysis of the body weights of all 12 groups (6 handled and 6 nonhandled groups) using the SNK multiple comparison test revealed lack of significant difference within all groups (Fig 6). In addition, t-tests showed that comparison of experiments I and II between each group of male WT, female WT, male null and female null groups did not differ significantly. The evidence suggests that the handling of pups for behavioral analysis was not an important factor in regard to body weight.

### Experiment II. Growth of body and organs in *Gnrhr* null mice before puberty onset

**Fig 7:**
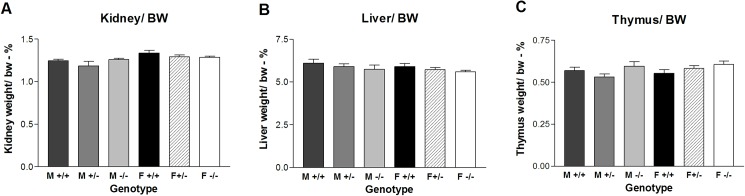
Growth of organs in a mouse line lacking the *Gnrhr* gene. The weight of the organs (grams) is shown as a percentage of body weight on postnatal day 28 for six groups of mice. The organs were (A) Kidney, (B) Liver and (C) Thymus. (F = female; M = male; +/+ = wildtype; +/- = heterozygous; -/- = null).

If the GnRH-GnRHR system has an extra-pituitary effect on early development during the first month of age, then the null group compared with the wildtype and heterozygous groups might show a difference in growth, which can be evaluated by organ weight in addition to body weight. The organ weights were determined only for the mice in experiment II as they were not handled. Kidney weight is considered to be one of the most stable measures of growth. The lack of a GnRH receptor in either males or females did not affect kidney weight as a ratio of body weight at 28 days of age (Fig 7A). There was a difference between the male het and female WT (SKN p<0.05) but this does not affect the null mice. The t-tests for kidney/body weight between male +/+ and -/- or female +/+ and -/- were not affected s(p>0.05).

The liver and thymus (each as a ratio of body weight) showed no significant difference among the six groups of pups at 28 days of age (Fig 7B and 7C). Thus, growth in terms of body or organ weight does not appear to be sensitive to a lack of GnRHR expression up to the age of 28 days.

### Experiments I/II. Metabolic changes (glucose) in *Gnrhr* null mice at 28 days

**Fig 8:**
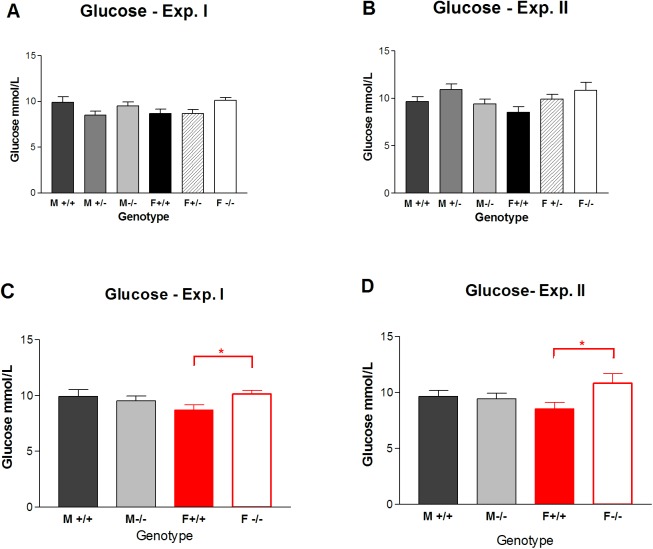
Comparison of glucose levels on 28 days in mice lacking the *Gnrhr* gene. Glucose is shown as mean ± SEM for six groups of mice. In experiment I mice were tested for sensory, motor and behavioral development from days 2 to 28, whereas in experiment II, mice were not tested. Statistics shown in the top row (A, B) are based on SNK multiple comparison tests; significant differences were not found within the six groups for either experiment I or II. In the bottom row (C, D) statistics are based on unpaired t-tests comparing only wildtype versus null separately for males and females. Red shows a significant differences in female +/+ versus -/- for both experiments I and II. Significant differences are shown as * (P> 0.05). (F = female; M = male; +/+ = wildtype; +/- = heterozygous; -/- = null).

Glucose was measured at day 28 in both experiments I and II. The means of the six groups were at the threshold of significance in that ANOVA was 0.048 for experiment I and 0.052 for experiment II. The SNK multiple comparison test was not significant for either experiment for the six groups (p> 0.05) (Fig 8A and 8B). However, the female null mice lacking the GnRH receptor had a significant increase in glucose when compared only with female wildtype mice (t-test p = 0.0257 for experiment I (Fig 8C) and p = 0.0379 for experiment II) (Fig 8D).

### Experiment II. Metabolic changes (inguinal adipose weight) in *Gnrhr* null mice at 28 days

**Fig 9:**
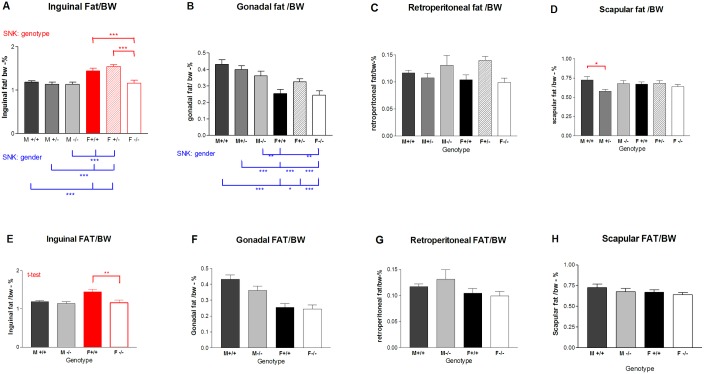
Comparison of fat pad weight at 28 postnatal days in *Gnrhr* gene knockout mice. The fat pad weight as a percentage of body weight is shown as mean ± SEM for six groups of mice. The fat pads are (A, E) Inguinal, (B, F) Gonadal, (C, G) Retroperitoneal and (D, H) Scapular. Statistics shown in the top row (A-D) were based on SNK multiple comparison tests in which red shows the significant differences among genotypes and blue lines show significant differences for sex. Statistics in the bottom row (E-H) are based on unpaired t-tests comparing only wildtype versus null separately for males and females; red is used to show significant differences. Significant differences are shown as * (P> 0.05), ** (P> 0.01) and *** (P>0.001). (F = female; M = male; +/+ = wildtype; +/- = heterozygous; -/- = null)

The most interesting result in the development of adipose tissue in pups was the significant decrease in the inguinal fat pad weight (per body weight) for the null females compared with wildtype and heterozygous females at 28 days (SNK p<0.001) (Fig 9A). Also, there were significant differences when comparing males with females, as the female (+/+ and +/-) inguinal fat pads/ body weight were larger than the three male groups (Fig 9A, SNK p<0.001). In contrast, the male gonadal fat pads/ body weight were significantly heavier than female equivalents (Fig 9B, SNK < 0.01 or 0.001 for M+/+ or M-/- vs. F+/+ or F-/-). The retroperitoneal (Fig 9C) and scapular fat pads/ body weight (Fig 9D) were not significantly different except that the male heterozygous mean was lower than the wildtype male mean for scapular fat. All values are presented as adipose tissues/body weight in Fig 9. Confirmation of the reduction in inguinal fat pad weight for female null mice compared with wildtype was confirmed by t-tests (p<0.01) (Fig 9E). However, individual t-tests of male wildtype versus male null or female wildtype versus female null mice did not reveal significant differences for gonadal, retroperitoneal and scapular fat pads (Fig 9F–9H).

### Experiment I. Metabolic changes (gonadal adipose histology) by sex in mice at 28 days

**Fig 10:**
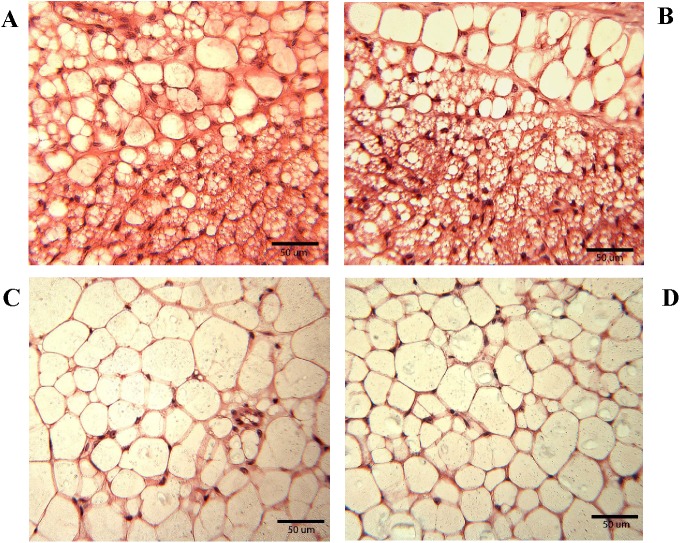
Comparison of histology for gonadal fat in a line of mice lacking the *Gnrhr* gene. Gonadal fat tissues were sampled at postnatal days 28–29, fixed in Bouin's solution and stained with hematoxylin and eosin for (A) female wildtype, (B) female *Gnrhr* knockout, (C) male wildtype and (D) male *Gnrhr* knockout. Nests of small adipocytes are present in both female genotypes, but rarely in males.

At 28 days of age, the gonadal adipose tissue in both wildtype and null females contained nests of small adipocytes along with medium and large adipocytes (Fig 10A and 10B). However, in wildtype and null males, medium and large adipocytes were dominant (Fig 10C and 10D).

## Discussion

To probe the role of GnRH in postnatal and prepubertal development, we examined mice in which the GnRHR was disrupted. The *Gnrhr* null pup provides a model in which both the direct and indirect effects of GnRH are blocked in their reproductive and non-reproductive functions during this period. The direct effects are blocked because the GnRH receptor is knocked out in all central and peripheral tissues and the indirect effects are blocked as the GnRHR is disrupted in the pituitary resulting in a downstream lack of sex steroids. An additional advantage of the *Gnrhr* knockout model is that sexual differentiation of the brain is permanently blocked; in the null male this occurs at birth, P0 [31], whereas in the null female, brain feminization occurs at P15-P22. Both losses are due to the lack of sex steroids following from the dysfunctional GnRHR. In contrast, *Gnrhr* null mice have gonadal differentiation and early stages of gonadal development as these events are activated before birth and are independent of GnRH action [40].

The present study demonstrates that regardless of sex or phenotype in *Gnrhr* wildtype, heterozygous or null mice, there is no significant difference in development of the motor, sensory or spatial orientation systems considered here. The implication is that the GnRH-GnRHR system, either directly or indirectly, is not essential for these non-reproductive aspects of development during the first month of life prior to puberty onset. Growth expressed as body weight was not affected at any time between 2 and 28 postnatal days in mice that had never been exposed to the effects of GnRH acting through its receptor. Growth of individual organs normalized to body weight, including kidney, liver and thymus, was not affected by the lack of the GnRH-GnRHR system in male or female mice. In addition, sexual differentiation of the brain in males or females is not involved in growth or the development of the motor or sensory systems tested. However, some alterations in metabolism occurred in *Gnrhr* null females; a significant reduction of inguinal fat pad weight relative to body weight was accompanied by an increase in glucose levels when tested at 28 days. This effect might be direct due to the loss of the GnRH receptor or indirect including the loss of estradiol between P15 and P28.

### Behavior

The behavioral tests were designed to include several tests for each evaluation of motor strength, locomotion, sensory development and spatial orientation. Thus the results in each category were confirmed by more than one type of test (except for hearing) and the order of the tests was designed to minimize stress to the pups.

Daily handling of nursing pups has the potential to be stressful to the pups and mother. In the present experiment, the non-handled and handled pups including wildtype, heterozygous, and null mice did not reveal any significant differences in body or organ weights or in glucose levels at the end of the experiment (28 days of age). We suggest that stress was minimized by removing only 4 out of 8 pups at a time and avoiding sound signals between mother and pups during testing. The heat lamp for the pups reduced hypothermic stress. Sex differences in response to stress have been reviewed [47], but were not apparent in our experiment.

### Growth

Growth in pups was not affected by the disruption of the *Gnrhr* gene. There is evidence that injection of GnRH in adult ewes can release GH from the pituitary [3], but lack of the GnRHR does not appear to alter GH in our experiments. The growth pattern of the pups in the present experiment is different from mice in which the growth hormone receptor (*Ghr-/-)* gene has been disrupted. Growth hormone deficiency did not affect growth until after two weeks of age in mice; the *Ghr*-/- mice had the same weight as WT mice at birth with the difference in weight becoming apparent only at about 3 weeks of age [48]. Further, the null mice weighed only about half of the wildtype mice by 4 weeks of age [49]. Our present results support normal growth hormone release during weeks 3 and 4 in the *Gnrhr* knockout line (+/+, +/-, -/-). We suggest that loss of the GnRH-GnRHR system either did not affect the neural or pituitary control of GH or was compensated by other systems. In summary, GnRH is not essential for body growth in developing mice between birth and one-month of age, regardless of sex or phenotype.

The kidney and liver were selected for evaluation as their weights tend to be stable in most circumstances. The thymus was selected for weight comparisons among the six groups as the thymus, which eventually disappears, could be an indicator of immunological status in the pups. However, the weight of the organs did not vary between groups in a significant way suggesting the GnRH system is not essential for growth of the body, liver, kidney or thymus between birth and prepuberty.

### Metabolics

The significant reduction in weight of the inguinal fat pads relative to body weight in 28-day-old female *Gnrhr* knockouts suggests GnRH might have a role during development in prepubertal mice. Fat deposits in different regions are known to vary individually depending on developmental stage, genetic background, sex steroids, insulin sensitivity and other factors [50]. The presence of *Gnrhr* -/- females with a decrease in inguinal fat depots suggests that genotype and sex are important in the inguinal subcutaneous region (Fig 9A and 9E). Variation in fat depots is based on adipocytes size, but also on preadipocyte replication and differentiation as shown by expression of biomarkers in individual depots [50, 51]. In the present experiment, a reduction in preadipocyte replication or differentiation/maturation is a possibility as these prepubertal mice had not yet been weaned at Day 28.

The option that GnRH has a direct effect on the inguinal adipocytes can be eliminated. Neither GnRH synthesis not GnRH receptors in adipose tissue have been reported, which implies that GnRH does not act directly on the peripheral fat pads.

As to indirect actions, GnRH is known to affect the metabolism of adipose tissue when its pituitary receptors are activated to release FSH/LH and secondarily sex steroids. Adipose tissue contains estrogen receptors ERα and ERβ and the antrogen receptor (AR). Our analysis shows that estradiol levels are very low in the *Gnrhr* null female mice based on the status of the reproductive organs [40]. Although estradiol has been measured throughout the prepubertal period in male and female rats [52], it has become apparent that low estradiol levels are difficult to measure accurately whether immunoassays or a combination of chromatography and mass spectrometry are used [38,39]. Nevertheless, it has been shown that wildtype female mice are exposed to estradiol beginning on about day P15. If a mouse is ovariectomized at this age or lacks the ability to convert testosterone to estradiol (aromatase KO), then female sexual behavior is lost as an adult [35–37]. The 28-day-old WT female mice in our colony were approaching puberty as vaginal opening normally occurs at 35 days of age [40]. Our *Gnrhr* null mice were deprived of the natural increase in estradiol between P15 and the end of the experiment at P28 [40]. This implies that the indirect effects of GnRHR disruption may be one factor in the decrease in female inguinal fat pad weight.

The males, however, did not have a decrease in the weight of their inguinal fat pads. One explanation is that wildtype males are not exposed to increasing testosterone (converted to estradiol) during the postnatal period from P2 to P28. The testosterone surge (1.6–1.7 ng/mL) at 1–2 hours after birth is followed by a drop in the level of testosterone (0.04 to 0.6 ng/mL) on days P2-P30 [31, 32, 34]. The increase in testosterone starts about P35 (0.9 ng/mL) and continues to rise by P40 (2.1 ng/mL), which is early puberty for males. Thus both the WT and *Gnrhr* null mice had little exposure to sex steroids during the 2-28-day period of examination. Also, the status of the inguinal fat pads at 28 days of life was clearly not affected by the lack of sexual differentiation of the male brain in the *Gnrhr* null mice [31].

Another indication that the GnRHR null mouse was not exposed to estradiol or testosterone is that the phenotype of the *Gnrhr* KO is very similar to the knockout models for estradiol and androgen receptors [53–56]. The βERKO and ARKO mice had milder changes in the ovary and testis compared with the αERKO and GnRHRKO, but both male and females were sterile or subfertile [55, 57–59]. These knockout models suggest that estrogen and testosterone actions are minimal in prepubertal mice, and the production of germ cells is arrested at an early stage.

To determine if the decrease in inguinal fat in the female is due only to lack of estradiol in the *Gnrhr* knockout female between P15 and P28, one can examine the effects of a tissue-specific knockdown of the ERα or AR only in adipose tissue. The results show that young mice (28 days old) have relatively minor effects, but full blown dysfunction occurs after puberty. In one study the αER expression was knocked down (ERα/Adiponectin Cre mouse) by 60% in adipose tissue for both males and females [60]. In WT and knockdown mice, the body weight of males or females did not differ in 28 day-old mice as in mice in the present experiment. Fat pad weight and metabolic factors were not measured [60]. Only in older mice at 12-weeks of age did the total body fat and gonadal pad weight increase significantly in females and glucose disposal become impaired in males [60].

A second study knocked down the androgen receptors (AR/aP2 Cre mouse) by 85–95% only in adipose tissue, but did not examine one-month old mice or females [61]. Changes in 12-week-old male mice included reduced body and gonadal adipose weight, but increased insulin blood levels and insulin resistance in the gonadal and subcutaneous tissue. As expected for reduced adipose weight, the adipocyte size did not increase in either gonadal or subcutaneous fat. Glucose tolerance was normal as were leptin transcript and plasma leptin levels; adiponectin and resistin transcripts were elevated. However by one year, the adipose-specific AR knockdown syndrome worsened in that mice had insulin deficiency but normal body weight [61]. Thus in both studies on adipose-specific loss of ERs or ARs [60, 61], mice do not become obese and only as adults show the full insulin dysfunction due to lack of sex steroids or their receptors.

In the present study, *Gnrhr* KO pups have reduced inguinal fat that is probably partially related to lack of estradiol prior to 28 days of age. They also have high resting levels of glucose, often a sign of insulin resistance. The question of whether these changes in adipose and glucose metabolism are transient depends on the metabolic profile in adults, In 4-month-old females of the same *Gnrhr* KO line, we found that not only glucose was significantly increased as in the pups, but also body weight and fat pad weights in these *Gnrhr* KO post pubertal mice. In older mice the tolerance tests revealed significant differences for males and females in regard to glucose metabolism and insulin sensitivity compared with WT and castrated mice (Busby and Sherwood, unpublished). In summary our evidence suggests that loss of the GnRH receptor at conception may indirectly, possibly by loss of sex steroids near puberty for females, affect specific fat depots in 28-day-old female mice. GnRHR disruption opens the possibility that the permanent loss of sex steroids and possibly other factors after puberty will result in continuing dysfunction in lipid, glucose and insulin metabolism.

Other factors in addition to sex steroids could affect the inguinal fat pad as there is a clear link between adipocyte metabolism and reproduction [62, 63]. For example, reproduction is sensitive to leptin feedback as a signal of the nutritional status and level of fat accumulation in the body. Leptin is released from adipocytes into the blood to activate their receptors within the brain; these receptors are not directly on GnRH or Kiss1 neurons, but on their afferent neurons [62]. Because GnRH is the final integrated signal for reproduction, a leptin signal to the brain would not enhance reproduction as the loss of the GnRHR in the pituitary of null mice would block gonadotropin release. Leptin blood levels are unlikely to be high as the body weight does not differ among genotypes. In fact, all nutritional signals (e.g. adiponectin, insulin and ghrelin) with receptors on the GnRH neurons or their afferent neurons [63] would be ineffective due to the impaired GnRH receptor. However, these nutritional hormones also have peripheral effects. Insulin resistance is implied by the high blood glucose in *Gnrhr* null pups; this suggests that a decrease in intracellular glucose and fat accumulation would occur in adipose tissue. It has been shown that estrogen deprivation in pregnancy leads to insulin resistance in a primate offspring; this may explain the phenomenon in the *Gnrhr* pups who are are born at an early stage of development and lack maternal or endogenous estradiol thereafter [64]. This could help to explain the connection between loss of estradiol and implied insulin resistance in young pups.

Finally, gonadal fat pads are also altered in females, but the difference from inguinal depots is that the histological appearance, but not weight differs. The weight of gonadal fat pads is not significantly different among female genotypes or among male genotypes, but the males have significantly higher weights relative to body weight than females (Fig 9B). Both WT and null females show the same histological pattern in gonadal fat, suggesting the difference from males is not due to loss of the GnRH receptor. Gonadal depots, unlike inguinal, are considered to be visceral fat. The male gonadal (aka epididymal) fat depots contained predominately large and medium sized adipocytes with a few smaller cells regardless of whether the males were WT or null (Fig 10). The histological appearance of the adipocytes was similar with those previously reported for 3-month old male mice [61]. However, the gonadal (ovarian) adipocytes in female mice (WT and null) were distinct from those of the males with pockets of tiny preadipocytes present in addition to areas of large and medium-sized cells. Either an additional stimulation for preadipocyte proliferation is present or differentiation is delayed in these 28-day-old females.

## Conclusions

During the first month of postnatal development, GnRH lacks a role in several non-reproductive processes including growth, motor functions, sensory detection and spatial orientation as assessed by behavior in a line of mice with disruption of the GnRH receptor. Stress effects due to daily handling of the pups did not produce an observable effect.

However, the data imply that GnRH has a role in postnatal metabolism as the *Gnrhr* null mice had a significant decrease in the subcutaneous (inguinal) fat pad and increase in glucose at 28 days of age, which is prepubertal for mice. The most likely explanation is that lack of estradiol in *Gnrhr* null females from P15-P28 may affect adipose metabolism. High glucose in the 28-day old null females may be related to insulin resistance resulting in lower intracellular adipose glucose and fat accumulation. These initial signs of metabolic effects in GnRHR disruption may foreshadow further dysfunction after puberty in adults.
